# Development of a Waterproof Crack-Based Stretchable Strain Sensor Based on PDMS Shielding

**DOI:** 10.3390/s18041171

**Published:** 2018-04-12

**Authors:** Seong Kyung Hong, Seongjin Yang, Seong J. Cho, Hyungkook Jeon, Geunbae Lim

**Affiliations:** 1 Department of Mechanical Engineering, Pohang University of Science and Technology (POSTECH), 77 Cheongam-Ro, Nam-Gu, Pohang 790-784, Korea; skhong@postech.ac.kr (S.K.H.); seongjin@postech.ac.kr (S.Y.); 2 School of Mechanical Engineering, Chungnam National University (CNU), 99 Daehak-Ro, Yuseong-Gu, Daejeon 305-764, Korea; scho@cnu.ac.kr

**Keywords:** crack-based stretchable strain sensor, waterproof sensor, PDMS shielding, underwater strain sensing

## Abstract

This paper details the design of a poly(dimethylsiloxane) (PDMS)-shielded waterproof crack-based stretchable strain sensor, in which the electrical characteristics and sensing performance are not influenced by changes in humidity. This results in a higher number of potential applications for the sensor. A previously developed omni-purpose stretchable strain (OPSS) sensor was used as the basis for this work, which utilizes a metal cracking structure and provides a wide sensing range and high sensitivity. Changes in the conductivity of the OPSS sensor, based on humidity conditions, were investigated along with the potential possibility of using the design as a humidity sensor. However, to prevent conductivity variation, which can decrease the reliability and sensing ability of the OPSS sensor, PDMS was utilized as a shielding layer over the OPSS sensor. The PDMS-shielded OPSS sensor showed approximately the same electrical characteristics as previous designs, including in a high humidity environment, while maintaining its strain sensing capabilities. The developed sensor shows promise for use under high humidity conditions and in underwater applications. Therefore, considering its unique features and reliable sensing performance, the developed PDMS-shielded waterproof OPSS sensor has potential utility in a wide range of applications, such as motion monitoring, medical robotics and wearable healthcare devices.

## 1. Introduction

Skin mountable or wearable electronic devices have recently grown in popularity due to their numerous advantages in the context of human–machine interactions [1,2,3,4,5,6,7,8,9,10,11,12,13,14,15,16,17,18,19,20,21,22,23,24,25]. A crucial component has been the development of flexible and stretchable strain sensors that have the ability to detect human motion when attached directly to the skin; this has been the focus of many studies [3,13,14,25].

A range of materials and structures have been investigated for use as flexible and stretchable strain sensors, including metallic thin film [1,2,3,4], nanoparticles [5,6], nanowires [7,8,9,10,11,12], carbon nanotubes [13,14,15,16,17,18,19], carbon black [20,21,22,23] and graphene [24,25]. Furthermore, significant research effort has resulted in the development of various flexible and stretchable sensors with excellent performance and features, especially in terms of their sensing range and gauge factor. The gauge factor describes how the change in relative resistance is dependent on strain. $\mathrm{GF}=(\Delta R/R_{off})/\varepsilon$, where R, $\Delta R=R_{on}-R_{off}$, and ε denote the resistance, change in resistance and applied strain, respectively.

Among the developed flexible and stretchable strain sensors, the crack-based sensor, which was inspired by the sensory system of a spider, shows very high sensitivity, and has the advantage of having a simple fabrication process involving metal deposition on a stretchable substrate, despite only having a narrow sensing range of 0–2% strain [1,2]. In previous research, an omni-purpose stretchable strain (OPSS) sensor which utilized a highly dense nano-cracking structure on a metal layer to overcome the limitations of a narrow sensing range was discussed [3]. Similar to previous bio-inspired stretchable strain sensors, the OPSS sensor has a very simple structure, consisting of a stretchable polyurethane (PU) membrane coated with a magnetron sputtered platinum (Pt) layer, where the cracking structure of the Pt layer was controlled to increase the sensing range. Due to its optimized and highly dense nano-cracking structure, the final OPSS sensor showed not only high sensitivity (with a gauge factor of ~30) but also a wide sensing range (up to 150% strain). The design also showed excellent linearity, high reproducibility and a fast response time, allowing for successful measurement of whole-body human motion at both the joint level (large displacement and high strength) and the skin level (small displacement and low strength).

Despite the improved performance of recently developed flexible and stretchable strain sensors, limitations remain with respect to utilizing the sensors in real-life applications. Performance degradation caused by a change in external environmental conditions, especially humidity [5,26,27], is a critical barrier to practical use. Because changes in humidity have a direct influence on the electrical properties of the conductive layer, an additional shielding process is essential for maintaining the sensing performance without altering the conductance.

This research investigated variations in the electrical properties of a previously developed nano-cracking structure-based OPSS sensor according to changes in humidity. The sensor was also modified by an additional stretchable layer of poly(dimethylsiloxane) (PDMS) to prevent variations in the electrical properties due to the shielding effect. The PDMS-shielded OPSS sensor showed very stable electrical characteristics, even with changes in humidity, and maintained a reliable sensing performance; however, the conductance of the original OPSS sensor was altered according to changes in relative humidity. Furthermore, it was observed that the PDMS-shielded OPSS sensor could be utilized even in underwater applications, as its conductive components were not exposed to the external environment. Considering its reliable sensing performance and watertight design, the PDMS-shielded waterproof OPSS sensor is suitable for real-life applications, in particular, motion monitoring systems.

## 2. Materials and Methods

### 2.1. Sensor Fabrication

**Figure 1:**
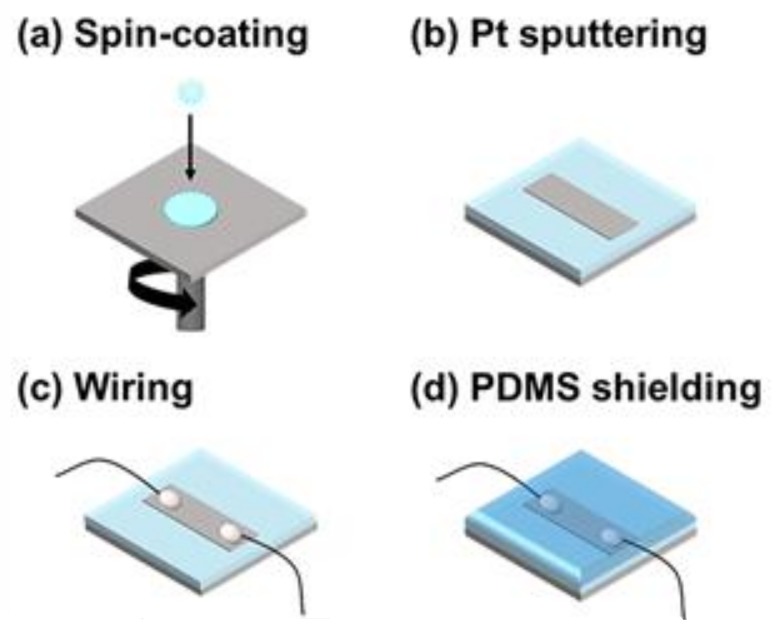
Fabrication of the poly(dimethylsiloxane) (PDMS)-shielded omni-purpose stretchable strain (OPSS) sensor. (**a**) Spin-coating of the polyurethane (PU) solution; (**b**) platinum (Pt) sputtering over a pattered film mask; (**c**) wiring process for measuring change in the resistance of the sensor; (**d**) shielding of the OPSS sensor with PDMS.

The fabrication of the sensor was based on the method developed previously for the OPSS sensor [3]. Figure 1a–d outlines the fabrication process. First, thermoplastic PU beads (Pellethane 2363-80AE; Lubrizol, Wickliffe, OH, USA) were dissolved in a mixture of tetrahydrofuran and dimethylformamide (60/40, *v*/*v*) to form a 10 wt % PU solution [3,28]. Then, the prepared PU solution was used to make a PU membrane by spin-coating on a glass slide, where the thickness of the membrane was controlled according to the spin speed (thickness of ~100 m achieved at 100 rpm). After cutting the membrane to a specific width, a thin layer of Pt was deposited on the membrane using a magnetron sputtering method, and electric wires were connected to both ends of the Pt-patterned PU membrane with silver epoxy (CW2400; CHEMTRONICS, Kennesaw, GA, USA). Finally, a layer of PDMS was cured on top of the OPSS sensor, resulting in the PDMS-shielded waterproof OPSS sensor.

### 2.2. Sensor Evaluation Setup

To evaluate the sensing performance, an experimental setup was developed involving strain application and conductance measuring, which were simultaneously controlled by a bespoke LabVIEW program; the setup was similar to that of previously published works [3,4,29]. A schematic of the experimental set-up is presented in the supplementary information (Figure S1). The evaluation method was as follows: the ends of the fabricated sensor were attached to a holder and a micro-translation stage (M-112; Physik Instrumente) to apply strain to the sensor by controlling the motion of the micro-translation stage. The current across the membrane was measured using a voltage source/measure unit (B2902A; Keysight)—a constant voltage was applied, the current was measured, and the resistance was calculated using Ohm’s law. Finally, the movement of the translation stage was controlled using the LabVIEW program to achieve the desired displacement, speed, and number of cycles. The applied strain and change in resistance of the sensor were monitored in real-time.

## 3. Results and Discussion

### 3.1. How Humidity Affects the OPSS Sensor

**Figure 2:**
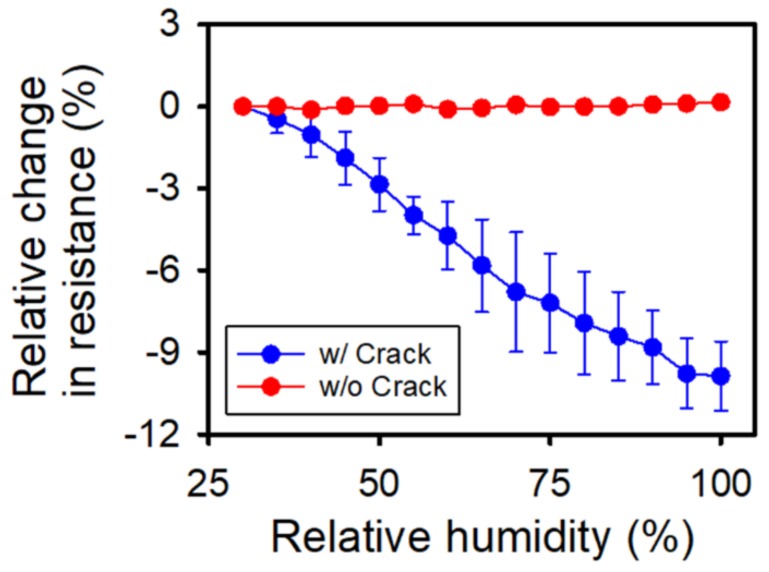
Relative changes in resistance due to the relative humidity for sensors without (red) and with (blue) crack formation.

First, the effect of humidity on the electrical conductivity of the previously developed OPSS sensor was investigated. We compared the resistance change of the OPSS sensor with crack and without crack in a humidity-changing environment. The OPSS sensor with crack was prepared by applying and releasing mechanical strain by up to 10% to achieve an even propagation of cracks among different OPSS sensor test samples. The OPSS sensor without crack was used as fabricated; it was not even detached from the glass slide, as detaching would generate mechanical strain causing the metal layer to crack. As shown by the blue line in Figure 2, the resistance of the OPSS sensor with the cracking structure decreased with increases in relative humidity, i.e., there was a 10% variation in the relative change in resistance at 100% relative humidity. However, changes in relative humidity did not always influence the resistance of the sensor. As seen in Figure 2, the resistance of the OPSS sensor did not change when the nano-cracking structure was not formed on the metal layer.

**Figure 3:**
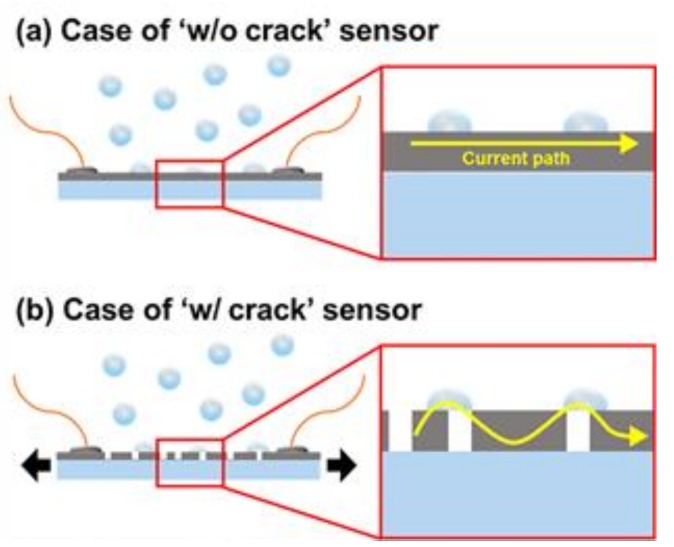
Schematic diagram of the electric current path for sensors (**a**) without (w/o) and (**b**) with (w/) crack sensors under high humidity.

The schematic depicted in Figure 3 illustrates why only the sensor with the nano-cracking structure showed a change in resistance. Figure 3a shows a schematic of an OPSS sensor to which no strain has been applied and which does not have the nano-cracking structure. The enlarged inset indicates that the conductivity of the metal layer remains intact, and condensed water droplets forming according to the high humidity do not affect the overall conductivity. However, when the nano-cracking structure is formed on the metal layer, as shown in Figure 3b, the condensed droplets from the high humidity create new pathways for the current to pass through, thus increasing the overall conductivity of the sensor.

**Figure 4:**
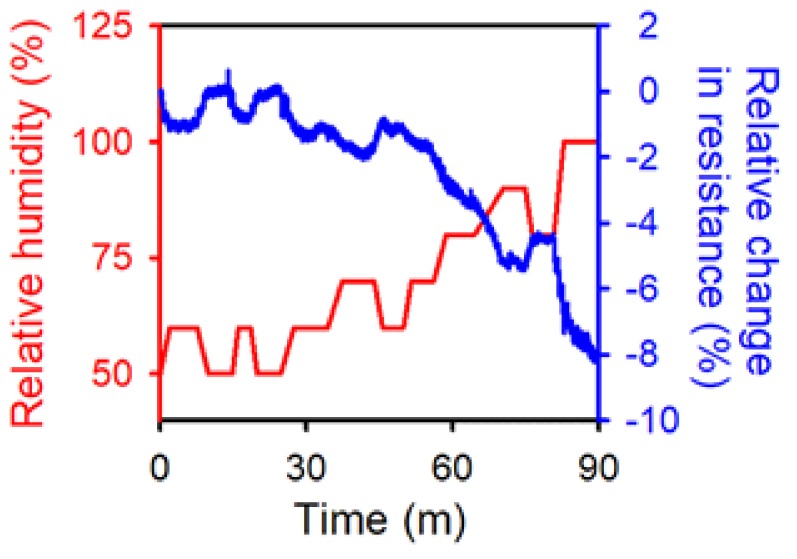
Relative change in resistance (blue) according to relative humidity level (red).

To further confirm how variation in the relative humidity affected the conductivity of the OPSS sensor, it was placed inside a humidity-controlled chamber, and the resistance was monitored as the relative humidity changed over time. The results are shown in Figure 4, where the blue line indicates the relative change in resistance of the OPSS sensor, and the red line indicates the relative humidity inside the chamber. The same trend is shown in Figure 2, where the resistance decreased as the relative humidity increased. On a different note, there is a continuous decrease in resistance in Figure 4 even after the relative humidity reaches 100%. The humidity control used in this experiment keeps blowing humid air into the chamber even after the relative humidity reaches 100%, and the chamber becomes oversaturated with humidity. Furthermore, as depicted in Figure 3, it is not the humidity that affects the resistivity of the sensor, but the condensed water droplets. Even after the relative humidity reaches 100%, the condensation of water droplets still takes place, opening up new pathways of conductivity. This explains why the resistance keeps drifting even after the relative humidity reaches 100%. However, the two graphs in Figure 4 show a consistent inverse correlation. Although the OPSS sensor as is would not be fit to be used as a humidity sensor, with further optimization of the crack formation and additional surface treatment properly selected to increase the sensitivity of the resistance change to the humidity of the environment, the OPSS sensor has the potential to be used as a humidity sensor.

### 3.2. Analysis of the Electrical Characteristics and Sensing Performance of the PDMS-Shielded OPSS Sensor

**Figure 5:**
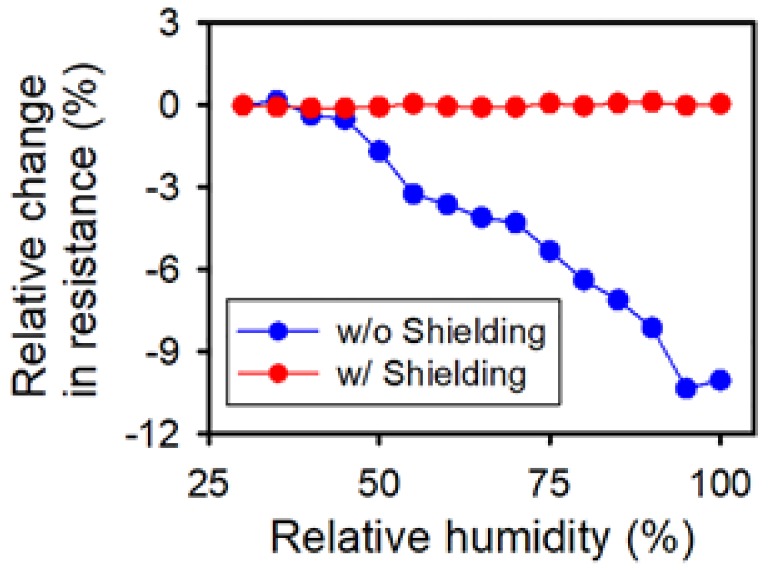
Relative change in resistance according to relative humidity for sensors without (blue) and with PDMS shielding (red). The blue dotted line here shares the same data with the blue dotted line in Figure 2.

To ensure the reliability of the OPSS sensor in environments with variable relative humidity, the sensor was covered with a layer of PDMS via the fabrication steps depicted in Figure 1a–d. The PDMS-shielded OPSS sensor was then placed within a humidity-controlled chamber alongside an unshielded sensor. The resistances of both sensors were measured during repeated humidity cycles. The results are shown in Figure 5, where the resistance of the PDMS-shielded OPSS sensor is indicated by the red line, and the resistance of the unshielded OPSS sensor is indicated by the blue line. The resistance of the PDMS-shielded OPSS sensor remained stable throughout the experiment. It can be concluded from these results that the PDMS layer covering the OPSS sensor successfully prevented environmental conditions from affecting the conductivity, thus reducing the reliability of the sensor.

**Figure 6:**
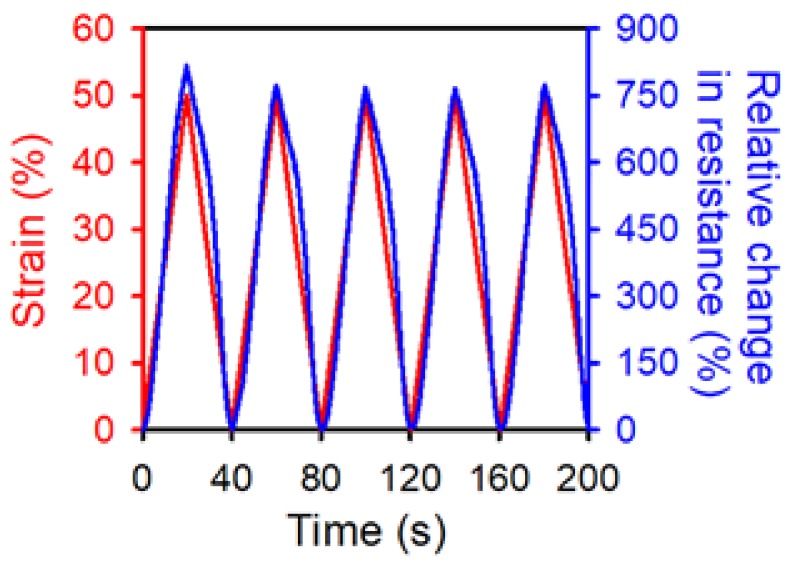
Relative change in resistance (blue) of the PDMS-shielded OPSS sensor during cycling at up to 50% strain (red).

The PDMS layer covering the OPSS sensor proved effective at shielding the sensor from humidity. However, further investigation is needed into whether the additional PDMS layer has any detrimental or beneficial effects on the sensing performance of the OPSS sensor. The strain cycle test was carried out for the PDMS-shielded OPSS sensor using the same testing set-up employed in previous work [3]. Figure 6 shows the relative change in resistance of the PDMS-shielded OPSS sensor during cyclic testing from 0% to 50% strain, and according to five repetitions. The relative change in resistance varied linearly depending on the strain applied to the membrane (up to ~50%), similar to the original OPSS sensor.

The gauge factor of the PDMS-shielded OPSS sensor can be derived from Figure 6 (Gauge Factor $=(\Delta R/R_{off})/\varepsilon$, where R, $\Delta R=R_{on}-R_{off}$, and ε denote the resistance, the resistance difference, and the applied strain, respectively). The gauge factor remained at ~18, which is still high enough to precisely measure various strains ranging from 0~50%. However, it is lower than the gauge factor of 30 of the original OPSS sensor. The PDMS-shielded sensor is less sensitive because the additional PDMS layer tightly holds the metal layer underneath in place, and this prevents the nano-cracking structure from propagating into larger cracks.

Although the PDMS layer reduced the gauge factor, it restored the sensor to its original resistance, even after multiple strain cycles. As was mentioned in the “Materials and Methods” section, the fabricated sensor was attached onto a micro-translation stage and went through strain cycles. After each cycle, the micro-translation stage returned to its original position. However, the resistance of the OPSS sensor did not fully return to its initial resistance but lingered above the initial resistance throughout the entire set of strain cycles [3]. The difference between the initial resistance and the resistance after a strain cycle is the hysteresis of the strain sensor. In previous work, the original OPSS sensor showed a hysteresis of 3–5% during the relaxation stage of the strain cycle, while the PDMS-shielded OPSS sensor had a hysteresis of ~0%. The PDMS-shielded layer provided excellent recovery characteristics, thus helping the sensor return to its original state; this resulted in enhanced hysteresis of the sensor. In summary, the PDMS-shielded layer provided the membrane with protection from humidity and improved the hysteresis at the cost of a decrease in the gauge factor; however, it remains more favorable than other stretchable strain sensors.

### 3.3. Underwater Strain Sensing of the PDMS-Shielded OPSS Sensor

**Figure 7:**
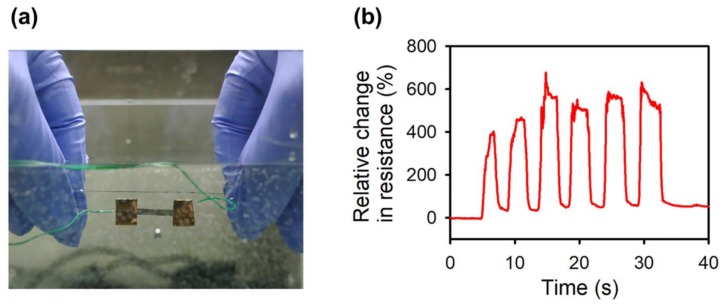
Underwater strain sensing test of the PDMS-shielded waterproof OPSS sensor. (**a**) Optical image of the underwater testing environment; (**b**) manually applied strain sensing test results for 6 strain cycles.

When the PDMS shielding is applied to the whole OPSS sensor, including its electrical connections, it can be used underwater. To reliably cover the whole sensor, two layers of PDMS were applied, at the top and bottom of the sensor. First, a thin PDMS layer was cured to the substrate at the location where the OPSS sensors to be placed. Another thin PDMS layer was then added to fully encapsulate the OPSS sensor in PDMS. There has been effort to cover a flexible sensor with a waterproof layer for functional sensing [30]. Packaging a stretchable strain sensor is a further step in the same direction. The packaging layers have to be stretchable and firmly adhered to the base material of the fabricated sensor throughout multiple cycles of stretching and shrinking. Figure 7a shows the PDMS-shielded waterproof OPSS sensor undergoing the manual strain cycle test underwater, (the results are presented in Figure 7b); the applied strain is ~5%, showing promising results for underwater strain sensing (See Supplementary Movie S1). Furthermore, since PDMS is a biocompatible material, the strain sensor can be implanted inside the human body to monitor internal deformations of human organs, such as the heart. Monitoring mechanical deformations inside the human body would provide more intuitive results compared to sensors that are limited to epidermal sensing.

## 4. Conclusions

In this work, a PDMS-shielded waterproof OPSS sensor impervious to changes in external environmental conditions, especially humidity, was developed. The results showed that the conductivity of the original OPSS sensor with a cracking structure increases as the surrounding humidity increases. This is due to the formation of condensed droplets under high humidity conditions, which creates new pathways for current to pass through. This humidity-dependent conductivity change indicates the potential application of OPSS as a humidity sensor. Through a simple additional fabrication process, the OPSS sensor was shielded by a PDMS layer so that the metal layer was isolated from the external environment. The developed PDMS-shielded OPSS sensor showed stable electrical characteristics regardless of change in humidity, while maintaining its strain-sensing performance. It showed improved hysteresis due to the recovery characteristics of the PDMS layer. Furthermore, the PDMS-shielded OPSS sensor could be utilized for underwater strain sensing due to the waterproofing characteristics of the PDMS over the conductive components of the sensor. Considering the improved sensing performance, the simple fabrication process and unique possibilities for underwater usage, the developed PDMS-shielded waterproof OPSS sensor could prove useful in various applications, including motion monitoring systems, medical robotics and wearable healthcare devices.

## Supplementary Materials

The following are available online at http://www.mdpi.com/1424-8220/18/4/1171/s1.

## References

[1] Kang D., Pikhitsa P.V., Choi Y.W., Lee C., Shin S.S., Piao L., Park B., Suh K.-Y., Kim T., Choi M. (2014). Ultrasensitive mechanical crack-based sensor inspired by the spider sensory system. Nature.

[2] Park B., Kim J., Kang D., Jeong C., Kim K.S., Kim J.U., Yoo P.J., Kim T. (2016). Dramatically Enhanced Mechanosensitivity and Signal-to-Noise Ratio of Nanoscale Crack-Based Sensors: Effect of Crack Depth. Adv. Mater..

[3] Jeon H., Hong S.K., Kim M.S., Cho S.J., Lim G. (2017). Omni-Purpose Stretchable Strain Sensor Based on a Highly Dense Nanocracking Structure for Whole-Body Motion Monitoring. ACS Appl. Mater. Interfaces.

[4] Jeon H., Hong S.K., Cho S.J., Lim G. (2017). Fabrication of a Highly Sensitive Stretchable Strain Sensor Utilizing a Microfibrous Membrane and a Cracking Structure on Conducting Polymer. Macromol. Mater. Eng..

[5] Lee J., Kim S., Lee J., Yang D., Park B.C., Ryu S., Park I. (2014). A stretchable strain sensor based on a metal nanoparticle thin film for human motion detection. Nanoscale.

[6] Chen S., Wei Y., Yuan X., Lin Y., Liu L. (2016). A highly stretchable strain sensor based on a graphene/silver nanoparticle synergic conductive network and a sandwich structure. J. Mater. Chem. C.

[7] Yao S., Zhu Y. (2014). Wearable multifunctional sensors using printed stretchable conductors made of silver nanowires. Nanoscale.

[8] Gong S., Lai D.T.H., Su B., Si K.J., Ma Z., Yap L.W., Guo P., Cheng W. (2015). Highly Stretchy Black Gold E-Skin Nanopatches as Highly Sensitive Wearable Biomedical Sensors. Adv. Electron. Mater..

[9] Amjadi M., Pichitpajongkit A., Lee S., Ryu S., Park I. (2014). Highly stretchable and sensitive strain sensor based on silver nanowire-elastomer nanocomposite. ACS Nano.

[10] Xiao X., Yuan L., Zhong J., Ding T., Liu Y., Cai Z., Rong Y., Han H., Zhou J., Wang Z.L. (2011). High-strain sensors based on ZnO nanowire/polystyrene hybridized flexible films. Adv. Mater..

[11] Hwang B.U., Lee J.H., Trung T.Q., Roh E., Kim D.I., Kim S.W., Lee N.E. (2015). Transparent Stretchable Self-Powered Patchable Sensor Platform with Ultrasensitive Recognition of Human Activities. ACS Nano.

[12] Ding Y., Yang J., Tolle C.R., Zhu Z. (2016). A highly stretchable strain sensor based on electrospun carbon nanofibers for human motion monitoring. RSC Adv..

[13] Yamada T., Hayamizu Y., Yamamoto Y., Yomogida Y., Izadi-Najafabadi A., Futaba D.N., Hata K. (2011). A stretchable carbon nanotube strain sensor for human-motion detection. Nat. Nanotechnol..

[14] Wang Z., Huang Y., Sun J., Huang Y., Hu H., Jiang R., Gai W., Li G., Zhi C. (2016). Polyurethane/Cotton/Carbon Nanotubes Core-Spun Yarn as High Reliability Stretchable Strain Sensor for Human Motion Detection. ACS Appl. Mater. Interfaces.

[15] Cai L., Song L., Luan P., Zhang Q., Zhang N., Gao Q., Zhao D., Zhang X., Tu M., Yang F. (2013). Super-stretchable, Transparent Carbon Nanotube-Based Capacitive Strain Sensors for Human Motion Detection. Sci. Rep..

[16] Cohen D.J., Mitra D., Peterson K., Maharbiz M.M. (2012). A highly elastic, capacitive strain gauge based on percolating nanotube networks. Nano Lett..

[17] Shin U.H., Jeong D.W., Park S.M., Kim S.H., Lee H.W., Kim J.M. (2014). Highly stretchable conductors and piezocapacitive strain gauges based on simple contact-transfer patterning of carbon nanotube forests. Carbon.

[18] Amjadi M., Yoon Y.J., Park I. (2015). Ultra-stretchable and skin-mountable strain sensors using carbon nanotubes–Ecoflex nanocomposites. Nanotechnology.

[19] Roh E., Hwang B., Kim D., Kim B., Lee N. (2015). Stretchable, Transparent, Ultrasensitive, and Patchable Strain Sensor for Human–Machine Interfaces Comprising a Nanohybrid of Carbon Nanotubes and Conductive Elastomers. ACS Nano.

[20] Kong J.H., Jang N.S., Kim S.H., Kim J.M. (2014). Simple and rapid micropatterning of conductive carbon composites and its application to elastic strain sensors. Carbon.

[21] Muth J.T., Vogt D.M., Truby R.L., Mengüç Y., Kolesky D.B., Wood R.J., Lewis J.A. (2014). Embedded 3D printing of strain sensors within highly stretchable elastomers. Adv. Mater..

[22] Mattmann C., Clemens F., Tröster G. (2008). Sensor for Measuring Strain in Textile. Sensors.

[23] Lu N., Lu C., Yang S., Rogers J. (2012). Highly sensitive skin-mountable strain gauges based entirely on elastomers. Adv. Funct. Mater..

[24] Jeong Y.R., Park H., Jin S.W., Hong S.Y., Lee S.S., Ha J.S. (2015). Highly Stretchable and Sensitive Strain Sensors Using Fragmentized Graphene Foam. Adv. Funct. Mater..

[25] Boland C.S., Khan U., Backes C., O’Neill A., McCauley J., Duane S., Shanker R., Liu Y., Jurewicz I., Dalton A.B., Coleman J.N. (2014). Sensitive, High-Strain, High-Rate Bodily Motion Sensors Based on Graphene–Rubber Composites. ACS Nano.

[26] Ryu H., Cho S.J., Kim B., Lim G. (2014). A stretchable humidity sensor based on a wrinkled polyaniline nanostructure. RSC Adv..

[27] Trung T.Q., Lee N.E. (2016). Flexible and Stretchable Physical Sensor Integrated Platforms for Wearable Human-Activity Monitoringand Personal Healthcare. Adv. Mater..

[28] Cho S.J., Nam H., Ryu H., Lim G. (2013). A Rubberlike Stretchable Fibrous Membrane with Anti-Wettability and Gas Breathability. Adv. Funct. Mater..

[29] Jeon H., Hong S., Cho S., Lim G. (2016). Development of an Integrated Evaluation System for a Stretchable Strain Sensor. Sensors.

[30] Dinh T., Phan H.-P., Qamar A., Nguyen N.-T., Dao D.V. (2016). Flexible and multifunctional electronics fabricated by a solvent-free and user-friendly method. RSC Adv..

