# Supplementary material for: Development of a Waterproof Crack-Based Stretchable Strain Sensor Based on PDMS Shielding
Source: Sensors (Basel). 2018 Apr 12;18(4):1171. doi: 10.3390/s18041171 (PMC5948561; doi:10.3390/s18041171)
Supplement: Supplementary file 1 [file sensors-18-01171-s001.zip › Supplementary Information.pdf]

# - Supplementary Information -

Development of a waterproof crack-based stretchable strain sensor based on PDMS shielding

Seong Kyung Hong, [a,†] *Seongjin Yang,* [a,†] *Seong J. Cho,* [b] *Hyungkook Jeon* [a,*] *and Geunbae Lim* [a,*]

[a]Department of Mechanical Engineering, Pohang University of Science and Technology (POSTECH), 77 Cheongam-Ro, Nam-Gu, Pohang 790-784, the Republic of Korea

[b]School of Mechanical Engineering, Chungnam National University (CNU), 99 Daehak-Ro, Yuseong-Gu, Daejeon 305-764, the Republic of Korea

Corresponding Author

*Geunbae Lim, Department of Mechanical Engineering, Pohang University of Science and Technology (POSTECH), 77 Cheongam-Ro, Nam-Gu, Pohang 790-784, the Republic of Korea, Tel. +82-54-279-2186, Fax +82-54-279-0479, e-mail: limmems@postech.ac.kr

*Hyungkook Jeon, Department of Mechanical Engineering, Pohang University of Science and Technology (POSTECH), 77 Cheongam-Ro, Nam-Gu, Pohang 790-784, the Republic of Korea, Tel. +82-54-279-2913, Fax +82-54-279-0479, e-mail: likeblue@postech.ac.kr

[†]These authors equally contributed to this work.

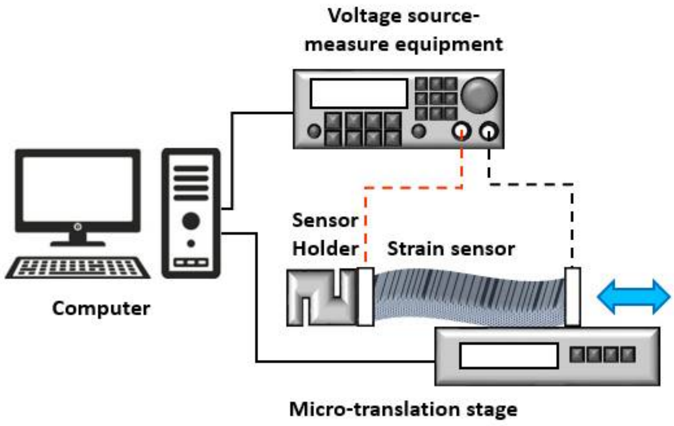

Figure S1. Experimental set-up for strain sensor evaluation.

The experimental setup comprises of a load cell, a micro-translation stage, a voltage source/measure unit. The ends of the fabricated sensor were attached to the sensor holder, and the micro-translation stage, respectively. The translational stage performed shuttle motions applying strain cycles to the mounted sensor. The load cell read the force applied during the cycles, and the voltage source/measure unit constantly applied voltage and measured the current to derive the resistance of the mounted sensor. All the equipment used in the set-up were controlled through our own LabVIEW code.

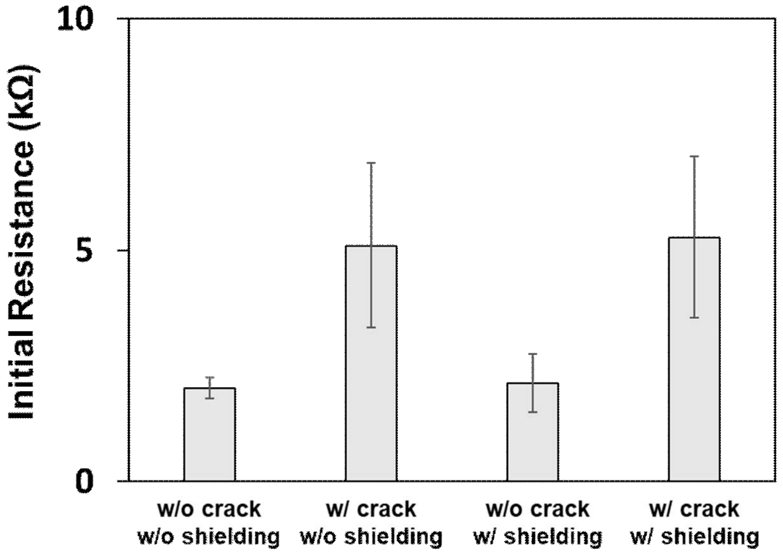

Figure S2. Comparison of the initial resistance of different types of OPSS sensor.

Figure S2 shows the comparison of initial resistance for four different OPSS sensor types, with two differing criteria: crack and shielding. It is clear from the graph that the initial resistance is increased when crack is formed on the metal layer of the sensor through detaching from the glass slide and deliberately applying strain of 10 %. On the other hand, PDMS shielding process does not affect the initial resistance as the initial resistances for the “w/o crack, w/o shielding sensor” and the “w/o crack, w/ shielding sensor” are the same, which also holds true for the “w/ crack, w/o shielding sensor” and the “w/crack, w/shielding sensor”.

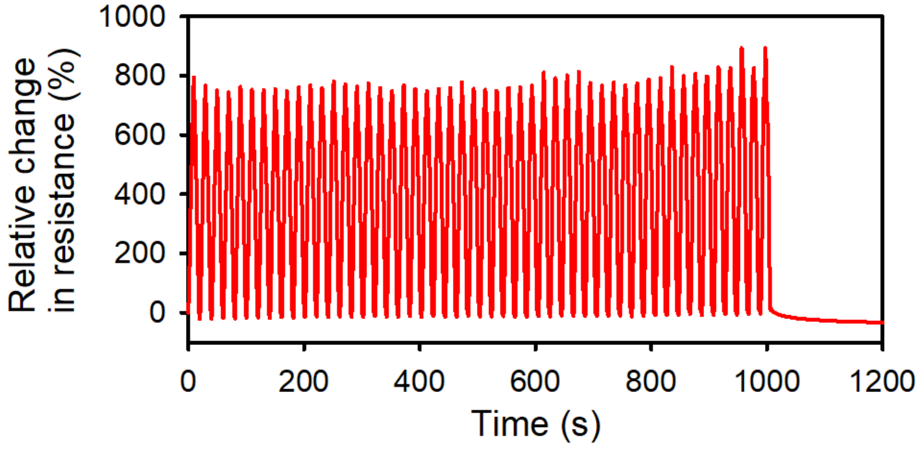

Figure S3. Repetitive strain cycle test up to 50 cycles of the PDMS-shielded OPSS sensor.

As shown in Figure S3, the performance of the sensor was kept fairly steady throughout the 50 cycles. Considering one of the biggest strong points of our sensor is that it is very easy to fabricate and thus disposable, this level of performance seems to be acceptable as the sensor can very easily be replaced by a new one.
